# Plasma Activated Water (PAW) in Organic Cultivation: An Experimental Study on Soil Properties and Plant Responses

**DOI:** 10.1111/1758-2229.70342

**Published:** 2026-05-05

**Authors:** Zakirul Islam, Tran Quoc Thinh, Hiroshi Hashizume, Masaru Hori, Motoki Kubo

**Affiliations:** ^1^ Department of Biotechnology, Graduate School of Life Sciences Ritsumeikan University Shiga Japan; ^2^ Center for Low‐Temperature Plasma Sciences Nagoya University Nagoya Japan

**Keywords:** bacterial biomass, nutrient cycling, organic cultivation, plant growth, plasma activated water

## Abstract

Plasma‐activated water (PAW), known for containing reactive oxygen and nitrogen species (RONS), is used to improve different agronomic traits in crops. However, its effects on soil microbial dynamics and plant growth under organic management remain underexplored. This study aimed to explore the effect of plasma‐activated water (PAW) on soil properties and the growth performance of spinach ( *Spinacia oleracea* L.) in an organic soil environment. The ultrahigh electron density nonequilibrium atmospheric–pressure plasma source (60 Hz–9 kV) was used to generate PAW in this experiment. Soils were treated with PAW at various dilution levels and assessed for microbial biomass, nitrogen mineralization, phosphorus circulation activities and bacterial community compositions using 16S rRNA gene sequencing. Plant growth parameters were also measured following cultivation under the same treatments. The results demonstrated PAW application significantly increased microbial biomass and enhanced nutrient cycling activities. 16S rRNA gene sequencing revealed the application of PAW influenced the indigenous bacterial community compositions. Moreover, PAW treatment also led to a notable improvement in plant growth parameters. Among the treatments, moderately diluted PAW yielded the most pronounced effects. These findings highlight the potential use of PAW to enhance soil fertility and plant growth in organic soil environments.

## Graphical abstract

This study demonstrates that PAW enhances microbial activity and nutrient cycling in organically managed soils, highlighting its potential for organic agricultural practices.

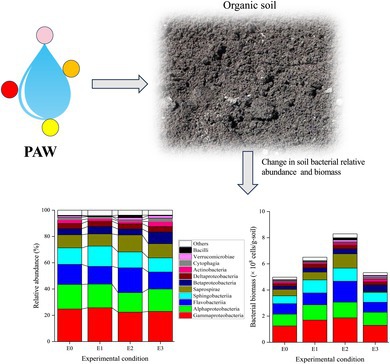

## 1 Introduction

In the atmosphere, lightning abiotically fixes nitrogen (N_2_) in the form of nitric oxide, which is then converted to biologically available forms (NH_4_^+^ and NO_3_^−^) (Yung and McElroy 1997; Navarro‐González et al. 2001). Accumulation of NH_4_^+^ and NO_3_^−^ in the soil after thunderstorm rainfall improves its fertility and subsequently increases crop yields (Mukherjee 1995; Cornell et al. 1988). However, atmospheric N_2_ fixation by lightning is an irregular occurrence and cannot be controlled based on demand. Industrial‐scale production of N_2_ fertilizers began in the early 20th century when the Haber–Bosch (H–B) process was approved for commercialization. The majority of synthetic N_2_ fertilizers supplied to the farmland are manufactured by the H–B process, which raises concerns due to their high energy demands and adverse environmental impacts (Kissel 2014).

Recently, cold (non‐thermal) plasma technology has been used to fix atmospheric N_2_ for fertilizers (Aceto et al. 2024). The cold plasma process consumes less energy than the H–B process (Rusanov et al. 1981). When plasma discharge interreacts with water, the plasma‐activated water (PAW) is generated, which contains different reactive oxygen and nitrogen species (RONS) (Antoni et al. 2025). The RONS include NO_3_^−^, which is primarily considered as N fertilizer in agricultural practices (Li Medrano et al. 2018). In addition to its reactive chemical species, PAW exhibits distinct physical characteristics, such as reduced surface tension and viscosity (Shaji et al. 2023). These changes are attributed to the disruption of hydrogen bonding resulting in breakdown of cluster formation in the water molecules. Such distinct physicochemical properties make PAW a promising alternative to conventional chemical fertilizers. For instance, PAW can be effectively used as a nitrogen source in agricultural applications (Park et al. 2013). Moreover, PAW can improve several agronomic traits, including seed germination, root development, plant defence responses and crop quality (Kučerová et al. 2019; Naumova et al. 2011; Ka et al. 2021; Sivachandiran and Khacef 2017). However, the impact of PAW on the properties of organic soil environment and plant growth performance remains poorly understood, highlighting the need for investigation to clarify its effects on organic cultivation practices.

In conventional agricultural systems, the chemical attributes of soil are given priority over the biological ones. In contrast, organic cultivation systems emphasize the importance of biological parameters such as soil organic matter content and microbial biomass and their activities to maintain soil fertility (Adhikari et al. 2014). The transformation of large and insoluble organic compounds into plant‐available nutrients is predominantly mediated by the soil microbial activities (Islam et al. 2023). To elucidate the effect of PAW application on the key biological indicators, including bacterial biomass and community compositions, nitrogen mineralization and phosphorus cycling activities were analyzed. Furthermore, the impact of PAW on the growth performance of spinach ( *Spinacia oleracea* L.) was evaluated under organically managed soil conditions with the aim of understanding its potential use in organic agricultural systems.

## 2 Methods and Materials

### 2.1 Preparation of PAW

The cold plasma irradiation conditions were based on a previously developed method (Hashizume et al. 2020). The ultrahigh electron density nonequilibrium atmospheric–pressure plasma source (60 Hz–9 kV), which produces electrons at an ultrahigh electron density (approximately 210^16^ cm^3^), was used for plasma irradiation (Iwasaki et al. 2008; Inui et al. 2010; Takeda et al. 2017). The water was irradiated for 5 min with Ar gas at a flow rate of 2 slm in open air. The values of pH, EC and NO_3_^−^–N were 3.3, 0.16 ds/cm and 16 mg/kg respectively.

### 2.2 Preparation of Organic Soil

Organic soil environment was prepared using a base soil with organic fertilizers. Base soil was prepared by mixing vermiculite, mountain soil (sand), black soil (andosol) and peatmoss (decomposed sphagnum moss) in a volumetric ratio of 5:3:1:1 respectively (Pholkaw et al. 2019). All materials were sourced from Kohnan Shoji Co. Ltd., Osaka, Japan. The fertilisation method from a previous study (cow manure 5%, oil cake 0.25%, soybean 0.25% and bone meal 0.05%, w/w) was employed to enrich the soil with essential nutrients (Pholkaw et al. 2019). Table 1 lists the physicochemical properties of the experimental soil.

**TABLE 1:** Physicochemical properties of the experimental soil.

Parameter	Value
TC (mg/kg)	41,800 ± 1140
TN (mg/kg)	1580 ± 110
TP (mg/kg)	1100 ± 54
TK (mg/kg)	10,700 ± 200
pH	6.7 ± 0.40
EC (ds/cm)	0.7 ± 0.01

Abbreviations: TC, total carbon; TK, total potassium; TN, total nitrogen; TP, total phosphorus.

### 2.3 Soil Incubation Experiment

The experiment was conducted at Ritsumeikan University, Kusatsu, Shiga, Japan (34°58′58.0″ N, 135°57′49.2″ E). Soil samples (500 g each) were treated with plasma‐activated water (PAW) at three concentrations: undiluted PAW (E1), 10‐fold diluted PAW (E2) and 50‐fold diluted PAW (E3). A control soil sample (E0) was treated with tap water. Initially, 150 mL of plasma‐activated water (PAW) was added to E1, E2 and E3, while 150 mL of tap water was added to E0 to adjust the soil water content to 30% for all treatments. All samples were incubated in Wagner pots at 23°C for 4 weeks. The soil water content was maintained at 30% throughout the incubation period by adding PAW to E1, E2 and E3 and tap water to E0 every alternate day. Samples were thoroughly homogenized prior to weekly analyzes. Soil bacterial biomass and inorganic N concentrations (NH_4_^+^–N and NO_3_^−^–N) were measured weekly. Phosphorus cycling activity and bacterial community compositions were assessed at the end of the four‐week incubation period. Three replicates were considered for each condition in this incubation experiment.

### 2.4 Analysis of Physicochemical Properties of Soil and PAW

Total carbon (TC) was analyzed using 0.5 g of soil with a Total Organic Carbon Analyzer (TOC‐VCPH, Shimadzu, Kyoto, Japan). Total nitrogen (TN), total phosphorus (TP) and total potassium (TK) were extracted from 1 g of soil using CuSO_4_·5H_2_O, H_2_SO_4_ and H_2_O_2_ at 420°C, following the Kjeldahl digestion method (Kjeldahl 1883). After extraction, the TN and TP concentrations were determined using a UV Visible spectrophotometer (U‐1900; Hitachi, Tokyo, Japan) following the indophenol and molybdenum blue methods (Nicholas and Nason 1957; Holman 1943), respectively. TK in the extract was measured using an atomic absorption spectrometer (Hitachi Z2300, Tokyo, Japan). Soil pH and EC were analyzed in a 1:2.5 ratio of soil and distilled water using a pH meter (LAQUA F‐72) and an EC meter (LAQUA EC‐33) (Horiba, Kyoto, Japan). The pH and EC of plasma‐activated water (PAW) were determined using the same instruments. All analyzes were conducted in triplicate.

### 2.5 Analysis of Soil Bacterial Biomass and Community Compositions

The bacterial biomass of soil was analyzed by quantifying the environmental DNA (eDNA) extracted using the slow‐stirring method (Aoshima et al. 2006). Soil samples (1.0 g) were mixed with 8.0 mL of DNA extraction buffer (100 mmol/L Tris–HCl (pH 8.0), 100 mmol/L sodium EDTA, 100 mmol/L sodium phosphate, 1.5 mol/L NaCl and 1% hexadecylmethylammonium bromide [CTAB]) and 1.0 mL of 20% (w/v) sodium dodecyl sulfate (SDS) solution. After agitation, 1.5 mL of the mixture was transferred to a microtube and centrifuged at 6000×*g* for 10 min at 20°C. The supernatant (700 μL) was collected and mixed with an equal volume of chloroform–isoamyl alcohol (24:1 v/v), followed by centrifugation at 18,000×*g* for 20 min at 20°C. The aqueous phase (500 μL) was transferred to a new microtube, mixed with 300 μL of isopropanol and centrifuged at 18,000×*g* for 20 min at 20°C. The resulting pellet was washed with 1.0 mL of cold 70% ethanol and centrifuged again at 18,000×*g* for 5 min at 20°C. Finally, the crude nucleic acid pellet was dissolved in 50 μL of TE buffer (10 mmol/L Tris, 1 mmol/L EDTA) after drying under reduced pressure to obtain the eDNA solution.

The extracted eDNA was quantified by measuring the intensity of the eDNA bands using agarose gel electrophoresis and analyzed with Kodak 1D 3.6 Image Analysis Software (Kodak, Rochester, NY, USA).

The bacterial eDNA extracted from soil was subjected to 16S ribosomal RNA (16S rRNA) gene sequencing. Sequencing was performed by Bioengineering Lab. Co. Ltd. (Kanagawa, Japan). PCR amplification targeting the V4 region was conducted using Bakt_341F and Bakt_805R primers sets. A library was prepared for each sample using the two‐step tailed PCR method. The purified DNA amplicons were sequenced in a 2 × 300 bp paired‐end run using a MiSeq system (Illumina, San Diego, CA, USA). Chimeric reads identified with USEARCH against the Greengenes 13_8 reference database were removed. The Operational Taxonomic Units (OTUs) table was clustered at a 97% similarity threshold using the Quantitative Insights into Microbial Ecology (QIIME, version 2) pipeline.

### 2.6 Analysis of Nitrogen (N) Mineralization

Nitrogen mineralization was evaluated by measuring the transformation of organic N into inorganic forms (NH_4_^+^–N and NO_3_^−^–N). Inorganic N in the soils was extracted with 1.0 M KCl in a 1:10 soil/solution ratio for 1 h. NH_4_^+^–N and NO_3_^−^–N in the extracts were determined by the indophenol blue method and brucine sulfanilic acid method respectively (Nicholas and Nason 1957). Analyzes of NH_4_^+^–N and NO_3_^−^–N were conducted in triplicate.

### 2.7 Analysis of Phosphorus (P) Circulation Activity

Organic P constitutes a large fraction of total soil P in agriculture and is microbially mineralized into plant‐available forms. A new method for the evolution of P circulation activity was developed in our previous study (Horii et al. 2013). This method is based on the microbial mediated formation of soluble P from organic P in soil. In this method, phytate (the most dominant form of organic P) is used as substrate. Briefly, 1 g of soil sample was added to a phytic acid solution (pH 7) containing 3.9 mg of P and then incubated for 3 days at 25°C. DW was used for the control. Water‐soluble phosphorus (SP) was extracted using 20 mL of DW and analyzed using the molybdenum blue method. The increase in the SP concentration reflected the P circulation activity. The following formula was used to calculate the P circulation activity, which was expressed on a scale of 0–100.

$$P\;\text{circulation activity}=\frac{\left(\mathrm{SP}\;\text{in}\;P3-\mathrm{SP}\;\text{in}\;P0\right)-\left(\mathrm{SP}\;\text{in}\;W3-\mathrm{SP}\;W0\right)}{\text{Total added}\;P}\times 100$$

where P0, P3, W0 and W3 represent the soil with phytic acid on Day 0, soil with phytic acid at Day 3, soil with DW at Day 0 and soil with DW at Day 3, respectively. Three replications were used to for the P circulation activity analysis.

### 2.8 Plant Cultivation Experiment

To evaluate the influence of PAW on spinach ( *Spinacia oleracea* L.) growth, a controlled plant cultivation experiment was conducted utilizing the same treatment regimes (E0, E1, E2 and E3) as described in the soil incubation experiment (Section 2.3). Organic soil was prepared according to the procedure detailed in Section 2.2. Each treatment was applied to 3 kg of prepared soil, adjusted to a 30% water content by adding 900 mL of PAW for the experimental treatments (E1, E2 and E3) and 900 mL of tap water for the control treatment (E0). The treated soils were thoroughly mixed and transferred into individual Wagner pots. Direct sowing was manually carried out in trays containing soil with 60% water content and incubated at 23°C at 12 h light/12 h dark photoperiod. One‐week‐old spinach seedlings were transplanted, with three seedlings Wagner per pot. Cultivation was performed under controlled environmental conditions, comprising a 12 h light/12 h dark photoperiod at a constant temperature of 23°C. Soil water content was maintained at 30% by watering on alternate days during cultivation period. The experiment was conducted for a period of 6 weeks, with all treatments replicated in triplicate.

The growth parameters including the shoot length, fresh shoot weight and fresh weight for all treatments were measured after 6 weeks of cultivation. The chlorophyll content in the leaves was analyzed as the average of five measurements taken at the same leaf position by a soil plant analysis development (SPAD) meter (SPAD‐502, Konica Minolta Sensing, Osaka, Japan).

The nitrate and antioxidant content of freshly harvested spinach leaves was analyzed to identify the effect of PAW on spinach quality. For these purposes, a mixture of 10 g fresh spinach leaves was prepared by adding 50 mL of distilled water and filtered through Whatman filter paper. The resulting juice was stored at −28°C until assayed within 1 week.

For the analysis of NO^−3^, 200 μL of spinach extract was mixed with 100 μL of brucine aminobenzenesulfonic acid solution (Jenkins and Medsker 1964). Then, 1 mL of diluted H_2_SO_4_ was added, and samples were kept in the dark at 4°C for 10 min. Next, 1 mL of DW was added to stop the reaction, and tubes were again placed at 4°C for 30 min. Finally, the absorbance was measured by a spectrophotometer at 410 nm.

The antioxidant capacity was analyzed according to the method described by Prieto et al. (1999). For the analysis, 100 μL of the same extract was used along with 1 mL of antioxidant solution (0.6 M sulphuric acid, 28 mM sodium phosphate and 4 mM ammonium molybdate). The mixture was then placed at 95°C for 90 min. The absorbance of the cooled solution was measured at 695 nm by a spectrophotometer and expressed as ascorbic acid equivalents (AAE).

### 2.9 Statistical Analysis

Statistical analyses were conducted using SPSS software (version 19.0; IBM Corp., Armonk, NY, USA). Analysis of variance (ANOVA) was employed to evaluate significant differences, followed by Tukey's post hoc test. Data visualization and graphical representations were generated using OriginPro (version 8.5; OriginLab Corporation, Northampton, MA, USA). Statistical significance was defined as *p* < 0.05.

## 3 Results

### 3.1 PAW Increases Soil Bacterial Biomass

**FIGURE 1:**
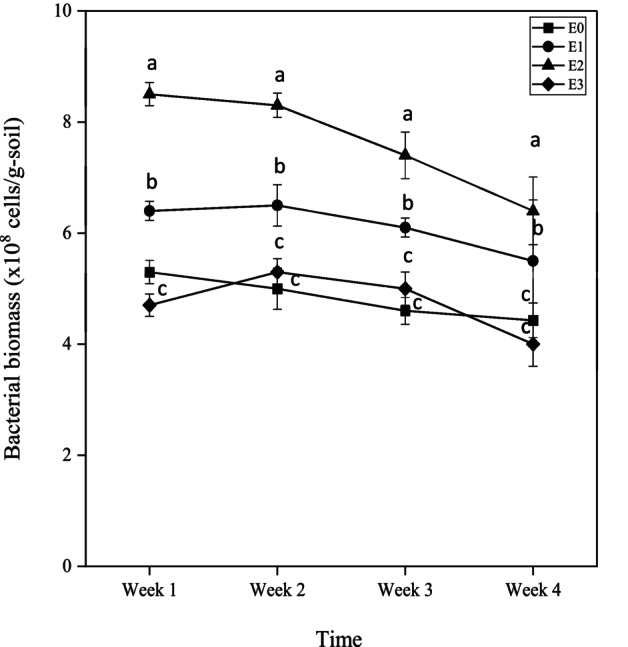
Temporal dynamics of soil bacterial biomass in organic soil following PAW treatments. The graphs show the changes in bacterial biomass in organic soil samples subjected to three concentrations of PAW treatments at four incubation periods. Data represent mean values ± standard deviation (*n* = 3). Data represent mean values ± standard deviation (*n* = 3). Different letters at the same time point between treatments indicate a significant difference (*p* < 0.05) based on ANOVA followed by post hoc analysis.

The impact of PAW on soil bacterial biomass was quantitatively assessed across three experimental conditions such as undiluted PAW (E1), 10‐fold diluted PAW (E2), 50‐fold diluted PAW (E3) and control condition (E0; tap water). As illustrated in Figure 1, bacterial biomass exhibited a significant increase in the PAW‐treated groups E1 and E2 compared to the untreated control (E0). Notably, the E2 treatment elicited the most pronounced response with bacterial biomass peaking at Week 2. A gradual decline in bacterial biomass was observed following Week 2; however, bacterial biomass in both E1 and E2 remained consistently higher relative to E0 and E3 throughout the experimental period. In contrast, the E3 treatment did not result in a significant change from the control, suggesting the condition of PAW applied in E3 were insufficient to enhance microbial activity.

These findings underscore the potential of PAW, particularly under the conditions represented by E2, to augment soil microbial biomass, thereby highlighting its influence on soil biological parameters.

### 3.2 Soil Bacterial Community Composition in Response to PAW Treatments

**FIGURE 2:**
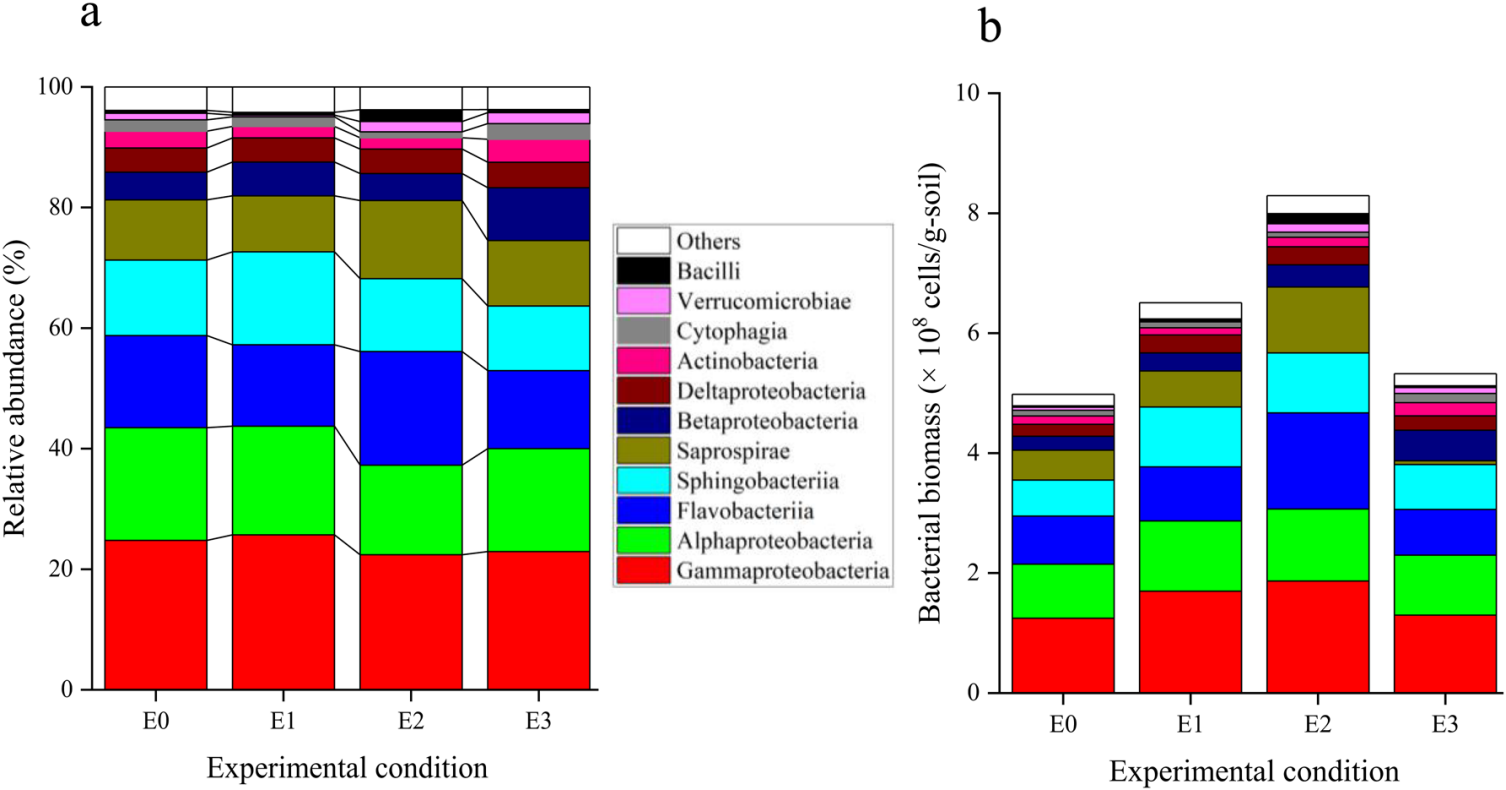
Changes in soil bacterial communities at the class level following 4 weeks of PAW treatment. The graphs show the changes in (a) relative abundance of dominant bacterial classes and their (b) biomass in organic soil samples subjected to three concentrations of PAW treatments over a 4‐week incubation period.

To evaluate the impact of plasma‐activated water (PAW) on soil bacterial communities, 16S rRNA gene sequencing was performed to analyze both the relative abundance and biomass of predominant bacterial taxa (Figure 2). In E2, the relative abundance of *Flavobacteria* and *Saprospirae* increased markedly following PAW treatment, whereas *Alphaproteobacteria* and *Sphingobacteriia* showed a notable decrease. In E1, PAW treatment resulted in a notable increase in the relative abundance of *Sphingobacteriia*, whereas the proportions of other bacterial classes remained comparatively stable across all treatment conditions. Furthermore, PAW application led to an overall increase in the biomass of the indigenous bacterial community, with the E2 treatment resulting in the most pronounced enhancement. These trends are further supported by data in Figure S1, which illustrates the relationship between the changes of the biomass of bacterial community and different PAW treatments.

At the genus level, 57 bacterial genera with relative abundance ≥ 0.1% were identified in control soils. PAW treatment induced changes in the relative abundance of several genera, with the most notable responses observed in E2. These community shifts are visualized in the polar heatmap presented in Figure S2.

### 3.3 Impact of PAW on Soil Nitrogen Mineralization

**FIGURE 3:**
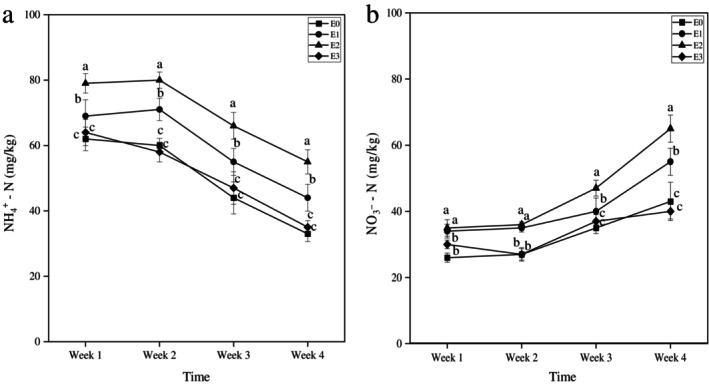
Temporal progression of N mineralization in organic soil following PAW treatments. The graphs show the changes in (a) NH_4_^+^–N and (b) NO_3_^−^–N concentrations in organic soil samples subjected to three concentrations of PAW treatments at four incubation periods. Data represent mean values ± standard deviation (*n* = 3). Different letters at the same time point between treatments indicate a significant difference (*p* < 0.05) based on ANOVA followed by post hoc analysis.

To elucidate the dynamics of nitrogen mineralization, temporal changes in the concentrations of ammonium (NH_4_^+^–N) and nitrate (NO_3_^−^–N) were quantified in soils (Figure 3). An increase in NH_4_^+^–N concentration was observed in treatments E1 and E2 relative to the control (E0) (Figure 3a). This accumulation of NH_4_^+^–N was subsequently followed by a gradual decline, concomitant with a progressive rise in NO_3_^−^–N levels across all treatments (Figure 3b). Treatments E0 and E3 exhibited comparable NH_4_^+^–N and NO_3_^−^–N concentrations throughout the experimental period.

The enhanced conversion of NH_4_^+^ to NO_3_^−^ in E1 and E2 implies a stimulation of ammonia‐oxidizing and nitrifying microbial populations, likely driven by the physicochemical properties of moderately diluted plasma‐activated water (PAW).

### 3.4 PAW Enhances P Circulation Activities

**FIGURE 4:**
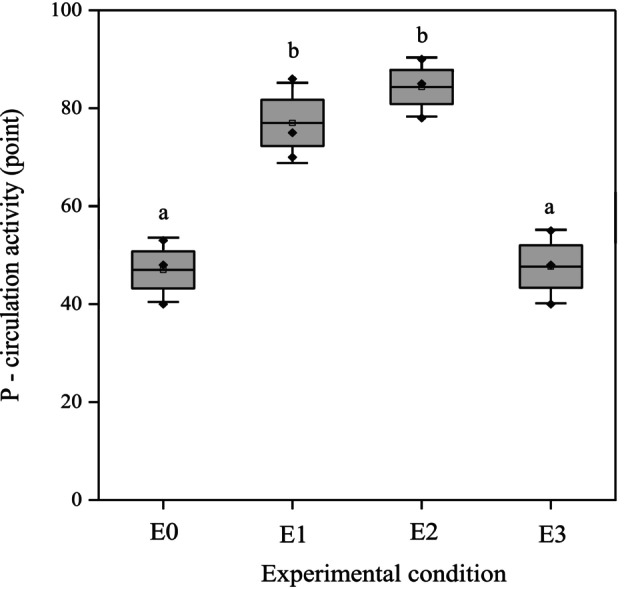
Effect of PAW treatments on phosphorus circulation activity in organic soil after 4 weeks of incubation. The graph shows the changes in phosphorus circulation activity in organic soil samples subjected to four concentrations of PAW treatments over a 4‐week incubation period. Data represent mean values ± standard deviation (*n* = 3). Different letters between treatments indicate a significant difference (*p* < 0.05) based on ANOVA followed by post hoc analysis.

Microbes mediated conversion of organic phosphorus to inorganic phosphorus was analyzed and considered to be phosphorus circulation activities in response to PAW treatments in organic soils (Figure 4). After 4 weeks of incubation, a significant increase in P circulation activity was detected in all PAW‐treated soils, with the exception of E3. Treatments E1 and E2 exhibited significantly elevated P circulation activity, reaching approximately 1.6 and 1.8‐fold increases, respectively, relative to the control (E0). In contrast, E3 did not differ significantly from the control, indicating that excessive dilution may attenuate the efficacy of PAW. These findings suggest that both non‐diluted and moderately diluted PAW effectively enhanced microbial processes involved in the mineralization of phosphorus, thereby contributing to improved P bioavailability in organic soils.

### 3.5 Impacts of PAW on Growth and Quality of Spinach

**FIGURE 5:**
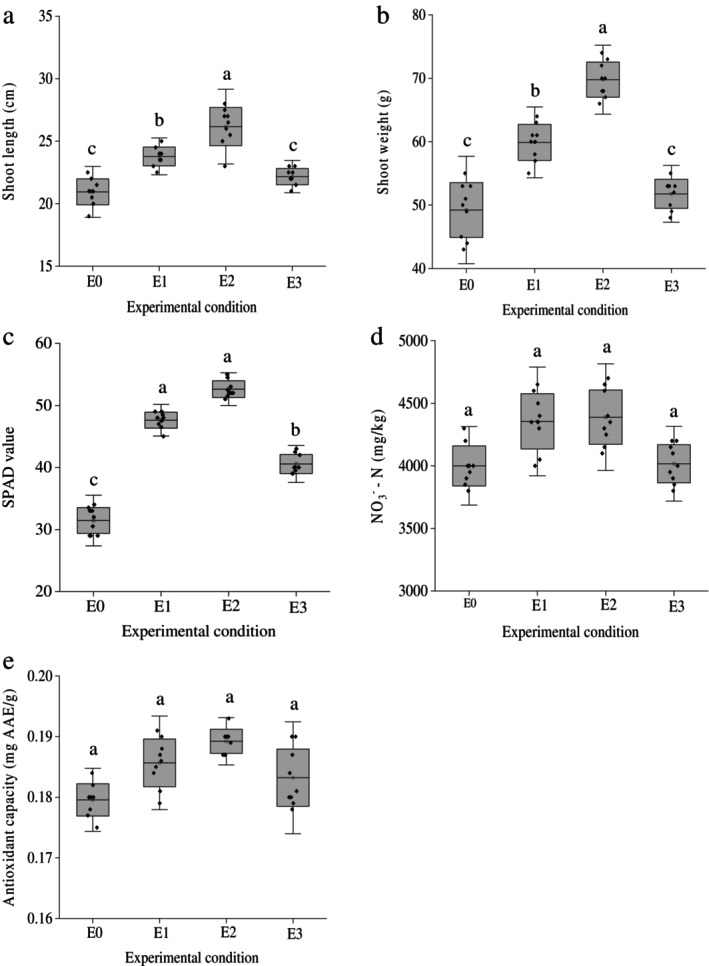
Effect of PAW treatments on growth parameters and quality indicators of spinach cultivated under an organic soil environment. The figures show changes in (a) shoot length, (b) shoot weight and (c) SPAD value, which significantly increased in E1 and E2 treatments compared to the control (E0). Quality indicators (d) nitrate and (e) antioxidant were not significantly affected by PAW application. AAE, ascorbic acid equivalent.

The effect of PAW on the growth parameters of spinach was evaluated by measuring shoot length, fresh shoot weight and SPAD value. All PAW‐treated soils (E1, E2 and E3) showed improvements compared to the control (Figure 5). The fresh shoot weight showed a notable increase in all PAW treatments (Figure S3). Compared to the control, the shoot length increased by 21%, 46% and 25% in E1, E2 and E3, respectively. Correspondingly, the fresh shoot weight increased by 32%, 36% and 10% in E1, E2 and E3, respectively. The SPAD value, indicating chlorophyll content, was also elevated in all PAW treatments, with E2 exhibiting the highest enhancement among the experimental groups. These results indicate that PAW positively influences spinach growth, particularly in the E2 treatment.

In addition to growth parameters, nitrate and antioxidant content were analyzed to evaluate the impact of PAW on the quality of spinach. Both parameters slightly, although not significantly, elevated in plants cultivated in PAW treated soil compared to plants from control treatment, suggesting PAW enhances spinach growth without adversely affecting the quality of spinach.

## 4 Discussion

In the present study, we investigated the effects of PAW on bacterial biomass, nitrogen mineralization and phosphorus cycling activity in organically managed soils. Bacterial biomass in the soil treated with PAW was consistently higher throughout the study period. The underlying mechanisms for bacterial biomass increase could be diverse. One possible explanation is that the chemical composition of PAW served as a nutrient source for promoting microbial proliferation. Moreover, PAW may indirectly influence microbial dynamics by modifying soil physicochemical properties. Previous studies show that plasma‐activated water (PAW) has lower viscosity and surface tension due to plasma‐induced disruption of hydrogen‐bonded water clusters (Yang et al. 2025). The reduced molecular clustering of PAW may improve infiltration and dispersion within soil pores, resulting in higher water retention capacity. Šimečková et al. (2020) demonstrated that PAW application can preserve soil porosity and enhance water retention by approximately 30% relative to conventional water. Such enhanced water‐holding capacity likely contributes to a favourable habitat for soil microorganisms, promoting sustained microbial proliferation (Bastida et al. 2008). The previous study on plant—microbe symbiotic interaction revealed PAW application significantly promoted AM symbiosis leading to increased fungal colonization (Binci et al. 2025).

PAW application enhanced N mineralization in organic soil. Organic N compounds, such as proteins, are insoluble and not directly available to plants (Islam et al. 2023). Their conversion into plant‐available forms like ammonium (NH_4_^+^) and nitrate (NO_3_^−^) through microbial mineralization is a considerable challenge in organic cultivation systems. The bacterial genera of proteobacteria are mainly responsible for this conversion in the natural environments (Koops et al. 1990; Head et al. 1993; Teske et al. 1994). In this study, 16S rRNA analysis of soil bacterial reveals PAW treatment increased Betaproteobacteria by 56% in E1 and 73% in E2 compared to control, likely contributing to enhanced nitrogen mineralization. Similar to N mineralization, PAW application enhanced P circulation in organically managed soil systems. This improvement is likely attributed to the stimulation of microbial communities involved in phosphorus mobilization. Soil microorganisms mediate the transformation of insoluble or organically bound phosphorus into bioavailable forms such as dihydrogen H_2_PO_4_^−^ and HPO_4_^2−^, which are readily taken up by plants (Yadav and Tarafdar 2001; Turner et al. 2002). In agreement with the previous study, the increase in relative abundance of several functional taxa of phosphate‐solubilizing bacteria including *Pseudomonas*, *Agrobacterium*, *Bacillus*, *Ralstonia*, *Burkholderia* and *Paenibacillus* in PAW treated soils could be reason for enhanced P circulation activities in this study (Figure S2) (Zhao et al. 2014; Chakraborty et al. 2009; Istina et al. 2015; Postma et al. 2010).

PAW application in organic soil environment improved growth parameters of spinach including shoot height, shoot weight and SPAD value. This increased growth is likely due to the enhanced nutrient circulation in soil together with additional nitrate supply by PAW. The increased spinach growth in this study aligns with the previous research reporting enhanced growth performance of spinach due to improved fertility following plasma irradiation in soil (Ketya et al. 2025). Moreover, enhanced growth performance has also been documented in tomato plants, where PAW functioned as an alternative nitrogen fertilizer (Priatama et al. 2025).

The application of PAW promotes an improvement in microbial‐driven nutrient circulation in organic soil environments. The observed results highlight PAW's potential role as a sustainable amendment for improving soil fertility in organic cultivation systems.

## 5 Conclusions

This study demonstrates that PAW can significantly enhance microbial activity, nutrient cycling and spinach growth in organically managed soils. These findings highlight the potential of PAW as a sustainable alternative to chemical fertilizers in agricultural practices. However, the experiment was conducted under incubation conditions; long‐term field studies are needed to confirm scalability. Future research should evaluate the efficacy of PAW on different plant species to support its adoption in organic cultivation practices.

## Author Contributions

**Zakirul Islam:** formal analysis and investigation, writing original draft. **Tran Quoc Thinh:** writing – review and editing. **Hiroshi Hashizume** and **Masaru Hori:** resources. **Motoki Kubo:** writing – review and editing and supervision.

## Conflicts of Interest

The authors declare no conflicts of interest.

## Supporting information


**Figure S1:** Principal component analysis (PCA) biplot illustrating the effect of PAW treatments on soil bacterial communities at the class level. The distribution of points indicates the variation in bacterial composition across treatments.
**Figure S2:** Heatmap showing the changes in relative abundance of soil bacterial genera following PAW application.
**Figure S3:** Influence of PAW treatments on spinach growth.

## Data Availability

The data that support the findings of this study are available on request from the corresponding author. The data are not publicly available due to privacy or ethical restrictions.

## References

[emi470342-bib-0001] Aceto, D. , P. F. Ambrico , and F. Esposito . 2024. “Air Cold Plasmas as a New Tool for Nitrogen Fixation in Agriculture: Underlying Mechanisms and Current Experimental Insights.” Frontiers in Physics 12: 1455481.

[emi470342-bib-0002] Adhikari, D. , T. Kai , M. Mukai , K. S. Araki , and M. Kubo . 2014. “Proposal for a New Soil Fertility Index (SOFIX) for Organic Agriculture and Development of a SOFIX Database for Agricultural Fields.” Current Topics in Biotechnology 8: 81–91.

[emi470342-bib-0003] Antoni, V. , E. Cortese , and L. Navazio . 2025. “Plasma‐Activated Water to Foster Sustainable Agriculture: Evidence and Quest for the Fundamentals.” Plants, People, Planet 7, no. 6: 1596–1603.

[emi470342-bib-0004] Aoshima, H. , A. Kimura , A. Shibutani , C. Okada , Y. Matsumiya , and M. Kubo . 2006. “Evaluation of Soil Bacterial Biomass Using Environmental DNA Extracted by Slow‐Stirring Method.” Applied Microbiology and Biotechnology 71: 875–880. 16518623 10.1007/s00253-005-0245-x

[emi470342-bib-0005] Bastida, F. , A. Zsolnay , T. Hernández , and C. García . 2008. “Past, Present and Future of Soil Quality Indices: A Biological Perspective.” Geoderma 147: 159–171.

[emi470342-bib-0006] Binci, F. , E. Cortese , E. Nouri , et al. 2025. “Plasma‐Activated Water Promotes and Finely Tunes Arbuscular Mycorrhizal Symbiosis in Lotus Japonicus.” BMC Plant Biology 25, no. 1: 544. 40281400 10.1186/s12870-025-06563-1 PMC12032643

[emi470342-bib-0007] Chakraborty, U. , B. N. Chakraborty , M. Basnet , and A. P. Chakraborty . 2009. “Evaluation of *Ochrobactrum anthropi* TRS‐2 and Its Talc‐Based Formulation for Enhancement of Growth of Tea Plants and Management of Brown Root Rot Disease.” Journal of Applied Microbiology 107: 625–634. 19426277 10.1111/j.1365-2672.2009.04242.x

[emi470342-bib-0008] Cornell, S. E. , T. D. Jickells , and C. A. Thornton . 1988. “Urea in Rainwater and Atmospheric Aerosol.” Atmospheric Environment 32: 1903–1910.

[emi470342-bib-0010] Hashizume, H. , H. Kitano , H. Mizuno , et al. 2020. “Improvement of Yield and Grain Quality by Periodic Cold Plasma Treatment With Rice Plants in a Paddy Field.” Plasma Processes and Polymers 1: 2000181.

[emi470342-bib-0011] Head, I. M. , W. D. Hiorns , T. M. Embley , A. J. McCarthy , and J. R. Saunders . 1993. “The Phylogeny of Autotrophic Ammonia‐Oxidizing Bacteria as Determined by Analysis of 16S Ribosomal RNA Gene Sequences.” Microbiology 139, no. 6: 1147–1153. 10.1099/00221287-139-6-1147 7689633

[emi470342-bib-0012] Holman, W. I. 1943. “A New Technique for the Determination of Phosphorus by the Molybdenum Blue Method.” Biochemical Journal 37, no. 2: 256–259. 16747628 10.1042/bj0370256 PMC1257890

[emi470342-bib-0013] Horii, S. , T. Matsuno , J. Tagomori , M. Mukai , D. Adhikari , and M. Kubo . 2013. “Isolation and Identification of Phytate‐Degrading Bacteria and Their Contribution to Phytate Mineralization in Soil.” Journal of General and Applied Microbiology 59, no. 5: 353–360. 24201147 10.2323/jgam.59.353

[emi470342-bib-0014] Inui, H. , K. Takeda , H. Kondo , et al. 2010. “Measurement of Hydrogen Radical Density and Its Impact on Reduction of Copper Oxide in Atmospheric‐Pressure Remote Plasma Using H2 and Ar Mixture Gases.” Applied Physics Express 3, no. 12: 126101.

[emi470342-bib-0015] Islam, Z. , Q. T. Tran , and M. Kubo . 2023. “Development of a Small‐Scale Cherry Tomato Cultivation Method Using Organic Soil.” Organic Agriculture 13, no. 2: 237–246.

[emi470342-bib-0016] Istina, I. N. , H. Widiastuti , B. Joy , and M. Antralina . 2015. “Phosphate‐Solubilizing Microbe From Saprists Peat Soil and Their Potency to Enhance Oil Palm Growth and P Uptake.” Procedia Food Science 1, no. 3: 426–435.

[emi470342-bib-0017] Iwasaki, M. , H. Inui , Y. Matsudaira , et al. 2008. “Nonequilibrium Atmospheric Pressure Plasma With Ultrahigh Electron Density and High Performance for Glass Surface Cleaning.” Applied Physics Letters 92, no. 8: 081503.

[emi470342-bib-0018] Jenkins, D. , and L. L. Medsker . 1964. “Brucine Method for the Determination of Nitrate in Ocean, Estuarine and Fresh Waters.” Analytical Chemistry 36: 610–612.

[emi470342-bib-0019] Ka, D. H. , R. A. Priatama , J. Y. Park , et al. 2021. “Plasma‐Activated Water Modulates Root Hair Cell Density via Root Developmental Genes in *Arabidopsis thaliana* L.” Applied Sciences 11: 2240.

[emi470342-bib-0020] Ketya, W. , N. N. Yu , T. R. Acharya , E. H. Choi , and G. Park . 2025. “Reduction of Microbial Load in Soil by Gas Generated Using Non‐Thermal Atmospheric Pressure Plasma.” Journal of Hazardous Materials 483: 136643. 39615386 10.1016/j.jhazmat.2024.136643

[emi470342-bib-0021] Kissel, D. E. 2014. “The Historical Development and Significance of the Haber Bosch Process.” Better Crops With Plant Food 98, no. 2: 31.

[emi470342-bib-0022] Kjeldahl, J. 1883. “New Method for Determination of Nitrogen in Organic Bodies.” Journal of Analytical Chemistry 22: 366–382.

[emi470342-bib-0023] Koops, H. P. , B. Böttcher , U. C. Möller , A. Pommerening‐Röser , and G. Stehr . 1990. “Description of a New Species of Nitrosococcus.” Archives of Microbiology 154: 244–248.

[emi470342-bib-0024] Kučerová, K. , M. Henselová , and K. Slováková Hensel . 2019. “Effects of Plasma Activated Water on Wheat: Germination, Growth Parameters, Photosynthetic Pigments, Soluble Protein Content and Antioxidant Enzymes Activity.” Plasma Processes and Polymers 16: 1800131.

[emi470342-bib-0025] Li Medrano, J. A. S. , V. Hessel , and F. Gallucci . 2018. “Recent Progress of Plasma‐Assisted Nitrogen Fixation Research: A Review.” PRO 6: 248.

[emi470342-bib-0026] Mukherjee, A. K. 1995. “Thunderstorm and Fixation of Nitrogen in Rain.” MASUAM 6, no. 1: 57–60.

[emi470342-bib-0027] Naumova, I. K. , A. I. Maksimov , and A. V. Khlyustova . 2011. “Stimulation of the Germinability of Seeds and Germ Growth Under Treatment With Plasma‐Activated Water.” Surface Engineering and Applied Electrochemistry 47: 63–65.

[emi470342-bib-0028] Navarro‐González, R. , C. P. McKay , and D. N. Mvondo . 2001. “A Possible Nitrogen Crisis for Archaean Life due to Reduced Nitrogen Fixation by Lightning.” Nature 412: 61–64. 11452304 10.1038/35083537

[emi470342-bib-0029] Nicholas, D. J. D. , and A. Nason . 1957. “Determination of Nitrate and Nitrite.” Methods in Enzymology 3: 981–984.

[emi470342-bib-0030] Park, D. P. , K. Davis , S. Gilani , et al. 2013. “Reactive Nitrogen Species Produced in Water by Non‐Equilibrium Plasma Increase Plant Growth Rate and Nutritional Yield.” Current Applied Physics 13: S19–S29.

[emi470342-bib-0031] Pholkaw, P. , A. Muraji , K. Maeda , et al. 2019. “Utilization of Wood Biomass for Organic Soil Based on the Soil Fertility Index (SOFIX).” Journal of Agricultural Chemistry and Environment 8, no. 4: 224.

[emi470342-bib-0032] Postma, J. , E. H. Nijhuis , and E. Someus . 2010. “Selection of Phosphorus Solubilizing Bacteria With Biocontrol Potential for Growth in Phosphorus Rich Animal Bone Charcoal.” Applied Soil Ecology 46, no. 3: 464–469.

[emi470342-bib-0033] Priatama, R. A. , H. K. Beak , S. Park , et al. 2025. “Tomato Yield Enhancement With Plasma‐Activated Water as an Alternative Nitrogen Source.” BMC Plant Biology 25, no. 1: 668. 40394503 10.1186/s12870-025-06701-9 PMC12090648

[emi470342-bib-0034] Prieto, P. , M. Pineda , and M. Aguilar . 1999. “Spectrophotometric Quantitation of Antioxidant Capacity Through the Formation of a Phosphomolybdenum Complex: Specific Application to the Determination of Vitamin E.” Analytical Biochemistry 269: 337–341. 10222007 10.1006/abio.1999.4019

[emi470342-bib-0035] Rusanov, V. D. , A. Fridman , and G. V. ASholin . 1981. “The Physics of a Chemically Active Plasma With Nonequilibrium Vibrational Excitation of Molecules.” Soviet Physics. Uspekhi 24: 447–474.

[emi470342-bib-0036] Shaji, M. , A. Rabinovich , M. Surace , C. Sales , and A. Fridman . 2023. “Physical Properties of Plasma‐Activated Water.” Plasma 6, no. 1: 45–57.

[emi470342-bib-0037] Šimečková, J. , F. Krčma , D. Klofáč , L. Dostál , and Z. Kazimova . 2020. “Influence of Plasma‐Activated Water on Physical and Physical–Chemical Soil Properties.” Water 12, no. 9: 2357.

[emi470342-bib-0038] Sivachandiran, L. , and A. Khacef . 2017. “Enhanced Seed Germination and Plant Growth by Atmospheric Pressure Cold Air Plasma: Combined Effect of Seed and Water Treatment.” RSC Advances 7: 1822–1832.

[emi470342-bib-0039] Takeda, K. , K. Ishikawa , H. Tanaka , M. Sekine , and M. Hori . 2017. “Spatial Distributions of O, N, NO, OH and Vacuum Ultraviolet Light Along Gas Flow Direction in an AC‐Excited Atmospheric Pressure Ar Plasma Jet Generated in Open Air.” Journal of Physics D: Applied Physics 50, no. 19: 195202.

[emi470342-bib-0040] Teske, A. , E. Alm , J. M. Regan , S. Toze , B. E. Rittmann , and D. A. Stahl . 1994. “Evolutionary Relationships Among Ammonia‐and Nitrite‐Oxidizing Bacteria.” Journal of Bacteriology 176, no. 21: 6623–6630. 7961414 10.1128/jb.176.21.6623-6630.1994 PMC197018

[emi470342-bib-0041] Turner, B. L. , M. J. Papházy , P. M. Haygarth , and I. D. Mckelvie . 2002. “Inositol Phosphates in the Environment.” Philosophical Transactions of the Royal Society of London. Series B, Biological Sciences 357, no. 1420: 449–469. 12028785 10.1098/rstb.2001.0837 PMC1692967

[emi470342-bib-0042] Yadav, R. , and J. Tarafdar . 2001. “Influence of Organic and Inorganic Phosphorus Supply on the Maximum Secretion of Acid Phosphatase by Plants.” Biology and Fertility of Soils 34, no. 3: 140–143.

[emi470342-bib-0043] Yang, T. , H. Zhang , Y. Bian , et al. 2025. “Ultrasonic‐Assisted Plasma‐Activated Water Extraction of Polysaccharide From *Hemerocallis citrina* Baroni: Structural Characterization and Antioxidant Mechanism In Vitro.” Food Chemistry 465: 142049. 39561598 10.1016/j.foodchem.2024.142049

[emi470342-bib-0044] Yung, Y. L. , and M. B. McElroy . 1997. “Fixation of Nitrogen in the Prebiotic Atmosphere.” Science 203: 1002–1004. 10.1126/science.203.4384.1002 17811121

[emi470342-bib-0045] Zhao, K. , P. Penttinen , X. Zhang , et al. 2014. “Maize Rhizosphere in Sichuan, China, Hosts Plant Growth Promoting *Burkholderia cepacia* With Phosphate Solubilizing and Antifungal Abilities.” Microbiological Research 169, no. 1: 76–82. 23932330 10.1016/j.micres.2013.07.003

