# Supplementary material for: Plasma Activated Water (PAW) in Organic Cultivation: An Experimental Study on Soil Properties and Plant Responses
Source: Environ Microbiol Rep. 2026 May 5;18(3):e70342. doi: 10.1111/1758-2229.70342 (PMC13139902; doi:10.1111/1758-2229.70342)
Supplement: Supplementary file 1 — Figure S1: Principal component analysis (PCA) biplot illustrating the effect of PAW treatments on soil bacterial communities at the class level. The distribution of points indicates the variation in bacterial composition across treatments. Figure S2: Heatmap showing the changes in relative abundance of soil bacterial genera following PAW application. Figure S3: Influence of PAW treatments on spinach growth. [file EMI4-18-e70342-s001.docx]

**Plasma activated water (PAW) in organic cultivation: An experimental study on soil properties and plant responses**

b

b

a

a

a *

Zakirul Islam, Tran Quoc Thinh, Hiroshi Hashizume, Masaru Hori**,** and Motoki Kubo

a

Department of Biotechnology, Graduate School of Life Sciences, Ritsumeikan University, Shiga, Japan

b

Center for Low-temperature Plasma Sciences, Nagoya University, Nagoya, Japan

**Corresponding author**

Motoki Kubo

Email: [kubo@sk.ritsumei.ac.jp](mailto:kubo@sk.ritsumei.ac.jp)


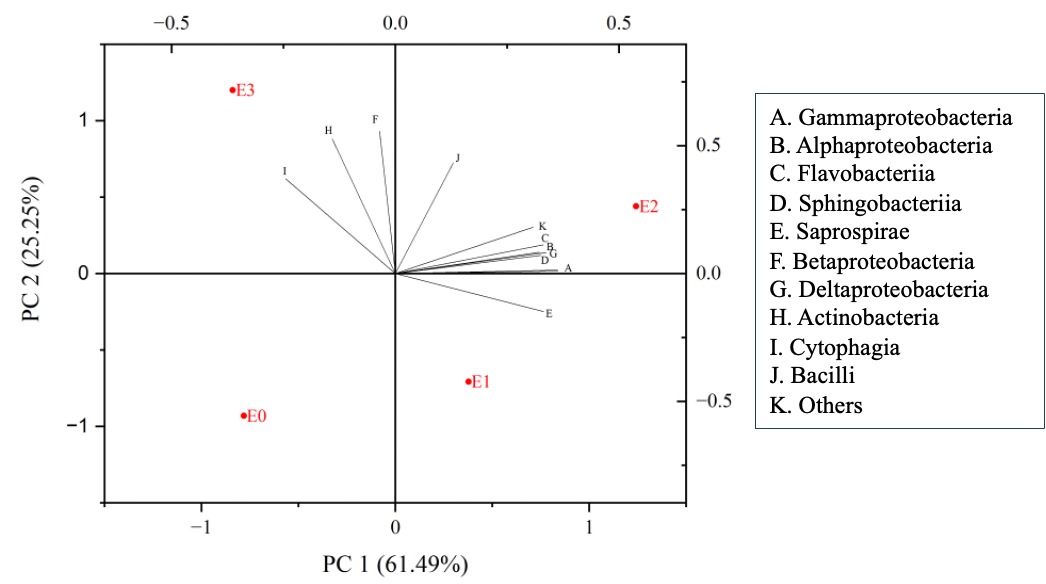


FIGURE S1 | Principal component analysis (PCA) biplot illustrating the effect of PAW treatments on soil bacterial communities at the class level. The distribution of points indicates the variation in bacterial composition across treatments.

**
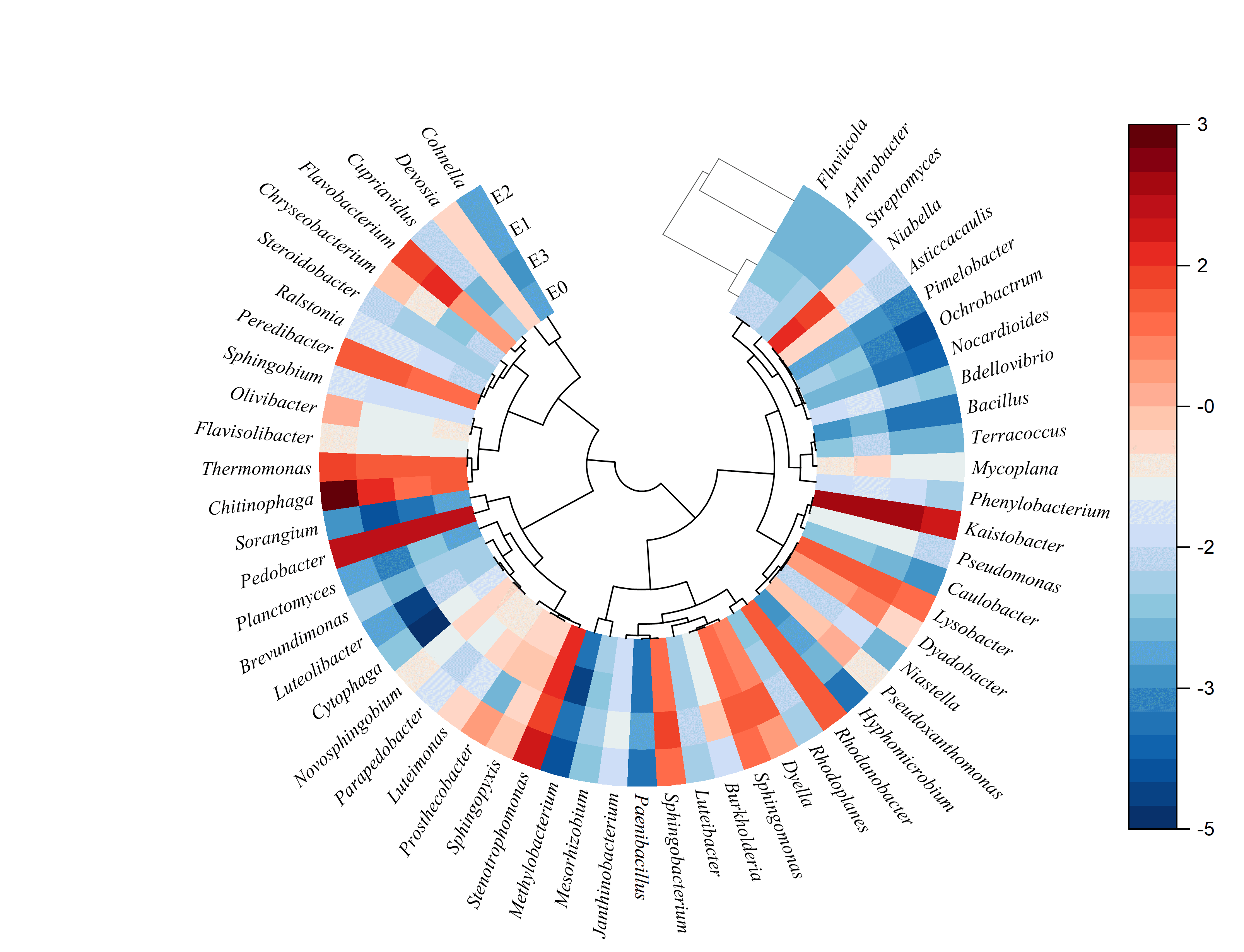
**

FIGURE S2 | Heatmap showing the changes in relative abundance of soil bacterial genera following PAW application.

Relative abundance values are log₂-transformed to enhance visualization of differences across treatments. Color gradients represent the variation in abundance levels in response to PAW treatments.


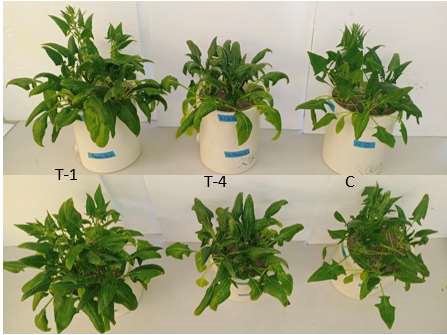

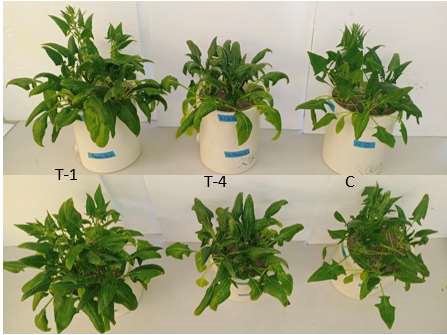

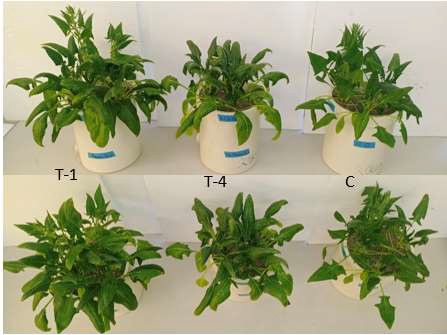

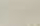

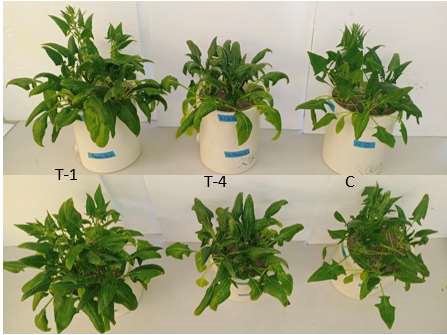


E0

E3

E2

E1

FIGURE S3 | Influence of PAW treatments on spinach growth.

Compared to the control (E0), all PAW-treated groups (E1–E3) showed increases in shoot length. Scale bar: 24 cm.
